# Restrictive feeding and excessive hunger in young children with obesity: A case series

**DOI:** 10.1002/ccr3.2411

**Published:** 2019-09-03

**Authors:** Sally G. Eagleton, Callie L. Brown, Melissa J. Moses, Joseph A. Skelton

**Affiliations:** ^1^ Center for Childhood Obesity Research Penn State College of Health and Human Development University Park PA; ^2^ Department of Nutritional Sciences Penn State College of Health and Human Development University Park PA; ^3^ Department of Pediatrics Wake Forest School of Medicine Winston‐Salem NC; ^4^ Department of Epidemiology and Prevention Wake Forest School of Medicine Winston‐Salem NC; ^5^ Brenner FIT^®^ (Families In Training) Program Brenner Children's Hospital Wake Forest Baptist Health Winston‐Salem NC

**Keywords:** appetite, case study, childhood obesity, family intervention, feeding practices, weight management

## Abstract

Treatment recommendations for childhood obesity include guidance to reduce portions and the consumption of high‐energy‐dense foods. These messages may unintentionally promote restrictive feeding among parents of children with obesity with excessive hunger. Clinical guidance may benefit from framing treatment messages to parents in the context of a nonrestrictive feeding style.

## 1 INTRODUCTION

Excessive child hunger is worrisome for parents of children with obesity. Parents often respond with restriction (controlling access/intake of certain foods), which can have an opposite effect (exacerbates weight gain). We present three cases where addressing restriction and implementing structured meals‐snacks contributed to improvements in child hunger and weight.

Parents play a key role in the prevention and treatment of childhood obesity,^1^, ^2^ particularly early in life when parents provide the contextual environment in which child food preferences and eating patterns develop.^3^ Parents use a variety of strategies to promote healthy child growth and dietary intake. To some degree, all parents of young children control what, when, and how much their child eats. There is considerable evidence that highly restrictive parent feeding practices in which parents control child access to and intake of certain foods while disregarding the child's preferences can contribute to overeating and higher body mass index (BMI).^4^, ^5^ Experimental studies have shown that restricting access to palatable “junk” foods increases children's preference for and consumption of restricted foods when freely available.^6^, ^7^, ^8^ There is also evidence that long‐term restriction can lead to Eating in the Absence of Hunger (EAH), a laboratory‐based behavioral measure of eating in response to food cues in the environment (ie, eating beyond satiety when presented with palatable foods).^9^ Three studies using the same longitudinal sample of girls showed positive associations between maternal restriction, EAH, and child weight status.^10^, ^11^, ^12^ Studies have shown that restrictive feeding is not only associated with higher child weight,^13^ but also with greater parental concern over child weight,^14^, ^15^ suggesting that parents may use restriction to help reduce energy intake to help slow weight gain. Though restrictive feeding practices are often well intentioned, restriction may actually contribute to children's preoccupation with food and problematic eating behaviors (eg, sneaking foods), ultimately exacerbating weight gain.

Clinicians struggle with how to assess young children with obesity whose parents report excessive child hunger. A child's large appetite could be related to a rare genetic condition such as Prader–Willi syndrome or a melanocortin 4 receptor mutation, which are associated with hyperphagia^16^ and the regulation of eating behavior,^17^ respectively. In contrast, reports of increased hunger may simply reflect normal changes in appetite associated with child growth and development. Based on our clinical assessment of parent feeding practices, mealtime rules, and family dynamics surrounding the home feeding environment (eg, mealtime conflicts), our clinic often considers the possibility that a larger‐than‐typical appetite and preoccupation with food may be a response to restrictive feeding in some cases of children with obesity. Treating children with obesity whose parents report excessive hunger is common within our tertiary care pediatric weight management clinic. It is challenging for parents when a child frequently complains of hunger; such behavior can cause stress within the family, conflict at mealtimes, and can increase the amount of food eaten between meals due to a child's constant requests for food.

In the following case series, we present observations from the evaluation and treatment of three young children with obesity in which parents reported concern over excessive child hunger and weight gain. The objectives of this case series are to: (a) document how parents report their children's complaints of excessive hunger within a clinical setting, (b) demonstrate how the implementation of a nonrestrictive, structured‐based approach to feeding^18^ can improve family feeding dynamics, and (c) provide evidence showing that childhood obesity treatment that includes a parent feeding component addressing restriction may contribute to improvements in children's eating behaviors and BMI.

## 2 CASE SERIES

The clinical observations for this case series took place at Brenner FIT^®^ (Families In Training), a tertiary care pediatric weight management clinic that sees 2‐ to 18‐year‐old children with obesity (BMI ≥ 95th percentile for age and sex). Treatment is interdisciplinary, involving a pediatrician, family counselor, dietitian, and either a physical therapist or an activity specialist and includes family‐focused goal setting, behavioral counseling, and individualized nutrition and physical activity education. Additional details regarding treatment at Brenner FIT have been previously published.^19^, ^20^, ^21^ Central to Brenner FIT's treatment philosophy is providing a parent education program that teaches a nonrestrictive, structure‐based feeding style, which is intended to give children autonomy over their food choices within a structured home feeding environment. To help families establish this feeding style, the program teaches Ellyn Satters' Division of Responsibility (sDOR).^22^ sDOR states that caregivers are responsible for providing structure by selecting what (ie, balanced meals and snacks), when (ie, predictable meal and snack times), and where (ie, eating together at the table) children eat. Children, on the other hand, are responsible for deciding whether and how much to eat from the food provided.^23^ Within the first month of treatment, a family counselor facilitates a group session in which parents are first exposed to the principles of sDOR. Over the course of the 6‐month treatment program, sDOR is reinforced in monthly visits with a family counselor and dietitian as well as at the intake and 6‐month medical review with a pediatrician. At Brenner FIT, patients are weighed at intake and at their 6‐month medical review in the same manner (light clothing and without shoes) using the same scale (Scale‐Tronix^®^ Model 5002) and stadiometer (Seca^®^ Model 240).

### 2.1 Patient 1

#### 2.1.1 Case history and evaluation

An 8‐year‐old white male who had normal development until age 5 when he began having seizures. Genetics and Neurology specialists had found a microdeletion on a gene associated with developmental delay, seizures, and hypotonia, but not hyperphagia or obesity. At age 3, his height‐for‐age was normal (75th percentile) but he was underweight with a BMI‐for‐age percentile < 5%. With the onset of seizures at age 5, he started to gain weight rapidly reaching the 50th percentile. He continued to gain weight rapidly and by age 8 his BMI was > 97th percentile (BMI 23.5, BMI *z*‐score 2.19), with height continuing to track at the 75th percentile. His mother reported excessive hunger with constant requests for second portions and snacks. Due to her concern for his overeating and rapid weight gain, he was referred to Endocrinology, but no underlying cause was found. His mother reported that he never seemed full and was often hiding, sneaking, or begging for food. His parents focused on the types of food he was eating, typically providing fruit to “fill him up,” which never seemed to work. There was also an effort to limit portions during meals. His parents served him with what they believed were normal portion sizes for his age and would make him wait 30 minutes before getting seconds and at least 30 minutes after dinner before allowing him to have a snack. In general, he skipped breakfast, since he was not hungry then and mornings were a struggle getting ready for school. Dinner was eaten as a family, and he typically consumed fruit after dinner and before bedtime.

#### 2.1.2 Treatment

In the clinic, we taught his mother the basics of healthy feeding dynamics incorporating principles of sDOR. As a first step, she lifted restriction at meals allowing him to eat until he was full, and provided more structure by scheduling snack times. Aside from scheduled meal‐snack times, she was encouraged to not respond to his food requests, but instead lead his attention to other activities. She expressed worry that he would “gorge” himself at meals if she implemented this guidance. Nonetheless, she agreed to put these principles into place.

#### 2.1.3 Outcome and follow‐up

Two months later, his mother reported successful implementation of the meal‐snack schedule. She was astonished that he did not eat as much as she thought he would at meals. He quickly stopped asking for an evening snack before bedtime, and she found that he was no longer sneaking foods. During this 2‐month time period, his BMI decreased to 22.8 and his BMI *z*‐score decreased to 2.07.

### 2.2 Patient 2

#### 2.2.1 Case history and evaluation

An 8‐year‐old Hispanic male who was referred to Brenner FIT due to ongoing weight gain, excessive appetite, and elevated triglycerides and liver enzymes. He had a chromosomal abnormality recently diagnosed that resulted in developmental delay and seizures, but had no known association with hyperphagia or obesity. He began gaining weight around 4 years of age, and with the support of the child's primary care pediatrician and a pediatric dietitian, his mother focused on increasing his acceptance of fruits and vegetables, limiting portion size, and improving her cooking habits. These changes were difficult to implement because of his picky‐eating (eg, rejection of fruits and vegetables), outbursts over food, and begging for snack foods.

#### 2.2.2 Treatment

Treatment involved implementation of a structured meal‐snack schedule in the home, allowing him to eat until full from the foods offered to him, and providing a wide variety of foods at meals, as his mother had eliminated many of his favorite foods to assist with weight management. This nonrestrictive feeding approach was reinforced in a follow‐up visit with the mother 2 months later, with additional guidance on meal planning.

#### 2.2.3 Outcome and follow‐up

His mother confidently instituted these principles and reported that he showed less anger at meal times, complained of hunger less often, and rarely begged for food anymore. Meals and snacks were no longer volatile, and the struggle over food decreased. His weight status had a remarkable response over 6 months, with his BMI decreasing from 24.4 to 22.5, and his BMI *z*‐score decreasing from 2.43 to 2.09.

### 2.3 Patient 3

#### 2.3.1 Case history and evaluation

A 7‐year‐old white female referred by her primary care provider for early‐onset severe obesity (BMI *z*‐score 2.7) and excessive hunger. Aside from having acanthosis nigricans and hypertriglyceridemia, she had no significant prior medical history. Her increasing weight began when she was about 4 years old. The family reported stress in the home, both around finances and parent relationships. She skipped breakfast, ate lunch at school, and the family ate dinner together at the table with no electronics or television. Her mother reported that she ate excessive amounts of food both after school and at dinner. Her “overeating” was her mother's biggest concern, saying she would eat until the point of vomiting, on average, four times a week. The family was always trying to slow down her speed of eating and get her to eat less, but typically allowed her seconds and thirds because of arguments and begging.

#### 2.3.2 Treatment

Her mother attended a Brenner FIT group class on sDOR with other parents that teaches parents how to institute a meal‐snack schedule and alternatives to restriction. The mother quickly implemented sDOR, not restricting portions and not commenting on the patient's eating.

#### 2.3.3 Outcome and follow‐up

Within a week, her mother reported that she ate less at dinner, left food on her plate, and her vomiting entirely resolved. This improvement was sustained between the class and her next visit a month later. Due to stress in the household, the family dropped out of the treatment program, thus follow‐up data on the patient's weight were not available.

## 3 DISCUSSION

In this case series, we demonstrate how parents may respond to a child with obesity that presents with excessive hunger by using restrictive feeding to slow weight gain. Our interdisciplinary team at Brenner FIT teaches families the principles of sDOR with the goal that parents will gain the necessary tools and self‐efficacy to institute a nonrestrictive structure‐based feeding style. Specifically, parents learn how to implement a consistent meal‐snack schedule and to allow the child to determine how much to eat from the food that is offered. The families in this case series successfully lifted restriction, and along with additional positive changes in family feeding dynamics, improvements in BMI were observed in two of the three patients with follow‐up weight data, and a partial or complete resolution of excessive hunger and other problematic eating behaviors (eg, sneaking food) was observed in all three patients. In addition to hyperphagia‐like symptoms that were observed in all three patients, two patients showed other signs (eg, hypotonia, seizure, and developmental delay) of a genetic abnormality. Although hyperphagia and weight gain were not the result of monogenic obesity (eg, Prader–Willi syndrome), it is possible that an underlying factor associated with certain genetic abnormalities contributes to weight gain and/or increases in appetite in early childhood. Additional work is needed that specifically examines this potential link.

This case series extends previous literature on the relationship between restrictive feeding and child weight.^6^, ^7^, ^8^ Studies have shown a positive association between restriction and weight,^24^ but have primarily been observational (relying on parent‐reported restriction) or laboratory‐based experimental studies. Randomized controlled trials that promote nonrestrictive feeding practices (ie, responsive feeding) have demonstrated success in reducing childhood obesity through 1‐3 years of follow‐up.^25^, ^26^, ^27^, ^28^ However, these are multi‐component interventions, and more research is needed to establish a causal link between parent feeding and obesity risk in children. Regardless, the improvements observed by our clinical program after families lifted restriction and increased structure are anecdotal evidence for the ecological validity of the empirical work to date.

This case series highlights important issues for health care providers to consider. Childhood obesity prevention and treatment recommendations include guidance to limit portion size and to reduce the consumption of high‐energy‐dense foods.^29^, ^30^ Parents may misinterpret these recommendations and institute restrictive feeding. For example, parents may completely eliminate foods considered “bad” or “unhealthy” from their child's diet^31^ or may limit the amount their child is allowed to eat. Following this guidance may be appropriate for most children given the positive link between both portion size and energy density with energy intake.^32^ However, these messages may contribute to a cycle of restrictive feeding and its unintended consequences among children with obesity with excessive hunger. It may be necessary to reevaluate expert committee recommendations such that clinical guidance on behavior change related to healthy eating and weight maintenance is framed in the context of a nonrestrictive feeding style. Additional training for pediatric health care providers and an increase in the number of clinics that specialize in family‐based childhood obesity treatment may also be warranted.

We believe that sDOR can contribute to positive outcomes for many children receiving clinical obesity treatment. However, a multidisciplinary intervention that incorporates sDOR may be most beneficial for 1) children with sudden weight gain and an increase in food‐seeking behaviors and 2) children from well‐functioning families marked by rules, routines, and positive communication. Restriction may be particularly high among parents with children that present with such symptoms, which may enhance the effectiveness of sDOR due to higher levels of baseline restriction. Further, it may be easier for well‐functioning families to adapt to the demands of sDOR, which requires some level of already established routines and overall healthy family dynamics.^33^ To help families effectively adapt to sDOR, Brenner FIT provides family therapy from licensed counselors trained in motivational interviewing,^34^ thus it is important for providers to keep this in mind when adopting a similar treatment approach. In conclusion, when a young child with obesity presents with parent reported excessive hunger, health care providers should consider exploring extrinsic causes that are amenable to treatment such as parents' use of restrictive feeding, as they also consider intrinsic etiologies.

## CONFLICT OF INTEREST

The authors have no conflicts of interest relevant to this article.

## AUTHOR CONTRIBUTIONS

SG Eagleton: Contributed to the analysis and interpretation of the data and was responsible for drafting the initial manuscript. CL Brown: Contributed to the analysis and interpretation of the data and revising the manuscript. MJ Moses: Contributed to the conception and design of this case series, the acquisition of the data, and revising the manuscript. JA Skelton: Contributed to the conception and design of this case series, the acquisition of the data, the analysis and interpretation of the data, and revising the manuscript. All authors read and approved the final manuscript.
